# The putative mechanisms underlying testosterone and cardiovascular risk

**DOI:** 10.12688/f1000research.3869.1

**Published:** 2014-04-04

**Authors:** Avinash Maganty, Jason R. Kovac, Ranjith Ramasamy

**Affiliations:** 1 Department of Urology, Baylor College of Medicine, Houston, TX, 77030, USA

## Abstract

The use of testosterone supplementation therapy (TST) is increasing primarily in men with symptomatic hypogonadism. While TST has been shown to have numerous benefits, as its use increases, the role on cardiovascular health must be explored. Previous evidence showed no adverse cardiovascular risks associated with TST use; however, more recent studies suggest that there may be an associated risk. The exact mechanism by which TST may contribute to cardiovascular risk has not been elucidated. Numerous mechanisms have been proposed which include testosterone’s effect on thromboxane A2 receptors, vascular adhesion molecule 1 receptors, erythropoiesis, and obstructive sleep apnea, all of which can ultimately lead to atherogenesis and increased cardiovascular risk.

Testosterone supplementation therapy (TST) is used to treat patients who suffer from symptomatic hypogonadism. The benefits of TST are numerous and include improved sexual function, bone mineral density, muscle mass and strength ^1^. Currently, there is no direct evidence linking TST with cardiovascular risk factors; however, several small studies have theorized potential mechanisms ( Figure 1) by which TST contributes to cardiovascular risk and these are summarized in this article.

**Figure 1.**
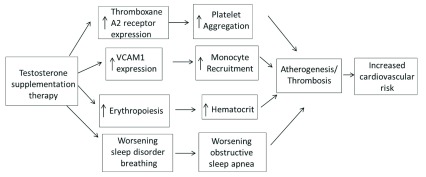
Proposed schematic by which TST may contribute to cardiovascular risk. TST increases TXA2 receptor expression, VCAM1 expression, erythropoiesis, and worsens sleep disorder breathing, all of which ultimately contribute to atherogenesis and worsening of cardiovascular health. VCAM1 - vascular cell adhesion molecule 1.

One proposed mechanism by which cardiovascular risk could be affected is in the regulation of platelet thromboxane A2 (TXA2) receptor expression by testosterone ^2^. TXA2, by acting on membrane receptors, helps in platelet aggregation and vascular smooth muscle contraction ^3^. TXA2 synthesis is increased in numerous thrombotic cardiovascular events ^4^. Ajayi *et al.* ^2^ examined the relationship between testosterone and TXA2 by measuring platelet TXA2 receptor density in response to TST in healthy men ^2^. Men were given testosterone cypionate 200 mg IM or placebo at day 1 and day 14 of the 10-week study period. Platelet TXA2 receptor density and dissociation constant then were measured. Testosterone supplementation was associated with increased TXA2 receptor density compared with placebo, suggesting that testosterone regulates the expression of platelet TXA2 receptors. The mechanism by which this occurs is not completely understood. However, it has been shown that inhibition of both transcription and translation of TXA2 attenuates testosterone’s effects on TXA2 receptor density in multiple cell lines ^5^. This finding suggests that testosterone may increase TXA2 receptor density by acting at the genomic level to stimulate its synthesis.

Testosterone has also been shown to contribute to atherosclerotic lesions by promoting monocyte adhesion ^6^. Atherosclerotic lesions are due to low density lipoprotein (LDL) infiltration into the arterial intima layer that subsequently results in endothelial cell activation and monocyte recruitment ^7^. The endothelial cells express vascular cell adhesion molecule 1 (VCAM1) which allows for monocyte adhesion before transmigration through endothelial junctions ^7^. These monocytes then release local inflammatory cytokines and metalloproteinases. The inflammatory cytokines promote smooth muscle proliferation that contributes to local plaque formation. Several studies have explored the role of dihydrotestosterone (DHT), a potent testosterone metabolite, in the various aspects of atherosclerotic plaque development ^8^. McCrohon *et al.* studied the effect of DHT in male umbilical vein endothelial cells ^9^. They demonstrated increased VCAM1 expression with increased monocyte adhesion. Similar findings have been observed using arterial endothelial cells ^9^.

Testosterone also plays a role in increasing hematocrit levels. Several studies have reported increased hematocrit levels following TST administration ^10, 11^. This correlation is likely to be secondary to the stimulation of erythropoiesis by testosterone. Increased hematocrit levels, or polycythemia, can itself contribute to adverse cardiovascular events. For example, an increased number of red blood cells can result in increased blood viscosity and predispose to thrombosis. A recent study by Marchioloi *et al.* analyzed the cardiovascular risks in patients with polycythemia vera ^12^. Patients, whose mean age was 64 (62% males), were divided into either a less intensive treatment group in which the target hematocrit was 45–50% or an intensive treatment group in which the target hematocrit was 45–50%. The choice of therapeutic approach was left to the investigator. The primary endpoint studied was the time until death from a cardiovascular cause or a major thrombotic event. The authors found that those whose hematocrit was maintained below 45% had significantly lower rate of cardiovascular death and major thrombotic events compared to those who had a hematocrit between 45–50% ^12^. Additionally, Kunnas *et al.* evaluated the association between hematocrit and coronary heart disease (CHD) in men over 55 ^13^. This study’s conclusions were similar to that previously discussed, in that men with a hematocrit greater than 50% were 1.8 times (1.1–2.7) more likely to die from CHD compared with men with a hematocrit of less than 50% after adjusting for coronary risk factors ^13^.

Testosterone may also contribute to cardiovascular disease by worsening pre-existing obstructive sleep apnea (OSA). Androgen deficiency is often observed in men who are obese or who have OSA ^14– 16^. Obese men have lower serum testosterone compared to age-matched non-obese men ^17^. This may be secondary to hypothalamic dysfunction or increased metabolic clearance as a result of central obesity ^17^. Similarly, previous studies have shown that men with OSA have low systemic testosterone levels, independent of increasing age or obesity, that correlate with the severity of hypoxia during sleeping hours ^16, 18, 19^. Additionally, the low systemic testosterone levels in these men have been shown to be reversible with nasal continuous positive airways pressure therapy (CPAP) ^16, 19^. The mechanism of reduced testosterone in men with OSA is likely to occur through OSA mediated dysfunction of the pituitary-gonadal axis ^17, 20^. Although controversial, TST has been used in men with severe OSA, given that men may remain androgen deficient if they are unable to comply with continuous positive airway pressure (CPAP) or lose weight ^21^. Hoyos *et al.* studied the effects of TST by conducting a randomized, double-blind, placebo controlled trial in 67 men with severe OSA. Testosterone treatment worsened the oxygen desaturation index and nocturnal hypoxemia compared to placebo, suggesting worsening of OSA ^14^. OSA itself is an independent risk factor for cardiovascular disease, and is associated with myocardial infarction and stroke ^22^. OSA likely contributes to disease by worsening atherosclerosis by affecting multiple atherogenic pathways such as hypertension, insulin resistance, dyslipidemia, endothelial dysfunction, and oxidative stress ^22^.

While testosterone has not been definitively linked to cardiovascular risk, these proposed mechanisms provide some insights into TST’s physiological effect on the cardiovascular system. Despite recent studies demonstrating an increased cardiovascular risk associated with TST ^23^, there is a large body of literature demonstrating the benefits of testosterone therapy. In fact testosterone replacement has demonstrated to decrease mortality ^24, 25^. Until the results of randomized trials are available, appropriate patient counseling and an emphasis on the importance of compliance with follow-up are essential prior to initiating testosterone replacement.
