# Supplementary material for: The putative mechanisms underlying testosterone and cardiovascular risk
Source: F1000Res. 2014 Apr 4;3:87. [Version 1] doi: 10.12688/f1000research.3869.1 (PMC3999929; doi:10.12688/f1000research.3869.1)
Supplement: Peer review history

# Peer review: Referee response for version 1

**DOI:** 10.5256/f1000research.4144.r4380

**Published:** 2014-04-22

**Authors:** Boback Berookhim

**Affiliations:** 1 Memorial Sloan Kettering Cancer Center, New York, NY, USA

Maganty and colleagues provide a timely and interesting report on a rather controversial topic which has been the subject of a number of recent, large, population-based, retrospective studies that have received media attention. The role of testosterone replacement therapy in worsening cardiovascular disease remains unanswered, and will likely require extensive, well-matched, randomized, placebo-controlled trials to prove its effect - whether protective, or aggravating - in cardiovascular disease.  

The authors, however, provide a well researched review of the putative mechanisms underlying this possible, though unproven, relationship. While the thromboxane A2, monocyte activation, polycythemia vera and obstructive sleep apnea pathways proposed are plausible, there is - to date - no clear evidence that any of these contribute to increased cardiovascular risk in men. Platelet activation and increasing atherosclerotic intimal plaque would be interesting topics for further research in testosterone replacement therapy, and will help to better elucidate this issue.

I have read this submission. I believe that I have an appropriate level of expertise to confirm that it is of an acceptable scientific standard.

# Peer review: Referee response for version 1

**DOI:** 10.5256/f1000research.4144.r4378

**Published:** 2014-04-15

**Authors:** Matthew S. Wosnitzer

**Affiliations:** 1 Department of Urology, New York Presbyterian Hospital, New York, NY, USA

This is a comprehensive summary of the potential mechanisms (i.e. platelet thromboxane A2 receptor, monocyte adhesion via endothelial cell vascular cell adhesion molecule 1, increased hematocrit level) underlying the possible association of testosterone supplementation therapy (TST) with cardiovascular outcomes. The discussion of possible mechanisms is important in light of recent questions about the cardiovascular side effects of TST, with studies showing both decreased and increased cardiovascular and mortality risk. An ideal study would be prospective and include additional measurements of hematocrit (or TXA2, VCAM1 for example) which could be related to cardiovascular outcomes and help to better select which men may derive the most benefit and least risk from TST.

Additionally, a recent by Yassin et al. ^1^ adds more data for the safety of TST (intramuscular testosterone undecanoate) in older men with late-onset hypogonadism and erectile dysfunction (mean age 59 years with mean treatment duration of over 4 years). No cardiovascular side effects were noted during the study and there was improvement of metabolic syndrome-related characteristics including obesity, blood glucose, cholesterol, and BP. Subjects were mostly were overweight or obese, and a minority had baseline cardiovascular comorbidities (12%), DM2 (31%) or HTN (45%) issues (which are similar to - or increased compared with - the baseline characteristics of the Finkle et al. study ^2^ – reference 23 in the article). In the future, more studies are needed to confirm these findings, especially investigating the mechanisms by which TST may affect cardiovascular outcomes.

I have read this submission. I believe that I have an appropriate level of expertise to confirm that it is of an acceptable scientific standard.

## References

[1] YassinDJDorosGHammererPGYassinAA: Long-Term Testosterone Treatment in Elderly Men with Hypogonadism and Erectile Dysfunction Reduces Obesity Parameters and Improves Metabolic Syndrome and Health-Related Quality of Life.*J Sex Med.*.2014; 8710.1111/jsm.1252324712761

[2] FinkleWDGreenlandSRidgewayGKAdamsJLFrascoMACookMBFraumeni JrJFHooverRN: Increased risk of non-fatal myocardial infarction following testosterone therapy prescription in men.*PLoS One.*.2014;9(1) : 87e8580510.1371/journal.pone.008580524489673PMC3905977

# Peer review: Referee response for version 1

**DOI:** 10.5256/f1000research.4144.r4383

**Published:** 2014-04-15

**Authors:** M. Emre Bakırcıoğlu

**Affiliations:** 1 Fulya IVF Center, Bahçeci Health Group, Istanbul, Turkey

This manuscript - *'The putative mechanisms underlying testosterone and cardiovascular risk'* - gives valuable information about the mechanisms of cardiovascular effects of testosterone supplementation therapy in hypogonadal men. This opinion article is well written and deserving of indexation.

I have read this submission. I believe that I have an appropriate level of expertise to confirm that it is of an acceptable scientific standard.
